# Assessment of Health Claims Related to Folic Acid in Food Supplements for Pregnant Women According to the European Regulation

**DOI:** 10.3390/nu13030937

**Published:** 2021-03-14

**Authors:** Laura Domínguez, Virginia Fernández-Ruiz, Patricia Morales, María-Cortes Sánchez-Mata, Montaña Cámara

**Affiliations:** Nutrition and Food Science Department, Pharmacy Faculty, Complutense University of Madrid (UCM), Plaza Ramón y Cajal, s/n, E-28040 Madrid, Spain; vfernand@farm.ucm.es (V.F.-R.); patmoral@ucm.es (P.M.); cortesm@farm.ucm.es (M.-C.S.-M.); mcamara@ucm.es (M.C.)

**Keywords:** food supplement, folic acid, pregnancy, food safety, health claims, nutrition

## Abstract

Pregnant women are a vulnerable group with increased nutritional requirements. The daily intake of folic acid, a crucial vitamin for embryonic development, must be reinforced through supplementation, as sometimes diets are not well equilibrated. As consumers increasingly rely on food supplements, it is vital to properly inform them about the health benefits provided by supplements’ consumption to ensure their safe use. The objective of this work was to assess the compliance level of health claims related to folic acid in food supplements commercialized in Spain according to the European regulation. Authors performed (1) a review of health-related claims approved for folic acid in Europe, (2) a market research of food supplements commercialized in Spain with those claims, and (3) a selection of food supplements for chemical analysis in the lab to assess these claims. The results showed that nine health-related claims are currently approved for folic acid in Europe. The analytical results for folic acid content in the selected samples were consistent with the declared values and within the tolerance ranges established in the European Guidance document. All samples included accurate dosages and met the legal requirements (European Regulations 1924/2006, 432/2012, 1169/2011) for all approved claims for folic acid.

## 1. Introduction

Food supplements are concentrated sources of nutrients (vitamins and minerals) or other substances (e.g., amino acids) with a nutritional or physiological effect, which are aimed at supplementing the normal diet for a specific period of time to reduce the risk of a specific disease [1,2,3].

The consumption of food supplements is increasing in Europe. In 2016, more than the 25% of the European population consumed food supplements to complement the habitual diet or maintain an adequate health status [4]. This percentage greatly increases up to 59.4% for pregnant women [5,6], a specific group of the population whose nutritional requirements (especially micronutrients such as folic acid) are increased [7,8,9]. According to the EuroPrevall Birth Cohort, a multi-center study carried out in nine European countries, the most commonly used food supplements by European pregnant women are those with folic acid, and Spain is the country with the highest consumption of food supplements (97.8% of Spanish pregnant women) [10,11].

### 1.1. Food Supplements’ Safety: An Overview of the State of the Art

The European Directive 2002/46/EC (last amendment in 2017) establishes a list of vitamins, minerals, and their sources allowed for use in food supplements [12], although safety dosage limits have yet to be established.

Food supplements could involve risks for consumer’s health. As an example, Brown and Wright (2019) carried out an extensive literature review focused on the key functions of folic acid during pregnancy as well as on the efficacy and safety of food supplements for pregnant women. The results concluded that high dosages (>1000 µg/day) of folic acid could provoke undesirable modifications in fetus neurodevelopment or even the appearance of side effects (diabetes, thyroid disorders, allergies, etc.) [13].

Food supplements are included in the Rapid Alert System for Food and Feed (RASFF) [14]. In the last five years (1 January 2015–31 December 2019), the RASFF received 848 notifications related to food supplements in Europe. The second cause of these notifications was the overdose of vitamins and minerals (87/463 notifications, that is, 18.8 %). Apparently there were no alerts regarding folic acid in the studied period [15].

### 1.2. Health-Related Claims of Food Supplements: Regulatory Framework in Europe and Its Implications for Consumers Health

Consumers should be properly informed about the health benefits provided by the consumption of food supplements in order to ensure their safe use [16,17,18,19]. According to the European Consumer Organization (Bureau European des Unions de Consommateurs, BEUC), one of the most important issues regarding consumers’ misunderstanding is related to the health claims made in food supplements’ labeling, presentation, and/or advertising [4,18].

Only those claims based on sound and independent scientific evidence are allowed to be included in food supplements labeling [20,21]. This ensures that all consumers (above all those considered the most vulnerable, e.g., pregnant women) can trust the health benefits claimed by these products and make well-informed food supplement choices [22,23,24]. Health claims referring to the reduction of disease risk and to children’s development and health must undergo a more complex assessment and authorization process. 

Spain counts on a Coordinated System of Fast Interchange of Information (SCIRI), a national network that ensures a constant vigilance of any risk related to food products that could affect consumer health. Within five years (1 January 2014–31 December 2018), the SCIRI has received several notifications regarding food supplements, and most of them were due to an incorrect labeling, including claims. In this five-year period, the notifications related to food supplements more than the doubled, increasing from 5.2% to 12.8%, [25].

Considering the worrying situation and the impact that it can have on consumers’ health and, most importantly, on at-risk groups of the population like pregnant women, the objective of the present work is to analyze and verify the level of compliance of the health claims related to folic acid in food supplements commercialized in the Spanish market according to the European regulation. To accomplish this goal, the authors performed the following tasks. First, a scientific literature review focused on the health-related claims approved for folic acid by the European regulation. Second, a market research of food supplements commercialized in the Spanish market with those health-related claims. Third, a selection of food supplements for pregnant women, which were subjected to subsequent laboratory chemical analysis to evaluate the appropriateness of the health-related claims included in their labeling, presentation, and/or advertisement.

## 2. Materials and Methods

### 2.1. Scientific Literature Review

In a first step, a detailed and in-depth study of the regulatory framework regarding health-related claims was conducted. An up-to-date review focused on the above-mentioned claims which are currently approved for folic acid was performed based on public information included in the European Register of nutrition and health claims made for food and food supplements (Available on: https://ec.europa.eu/food/safety/labelling_nutrition/claims/register/public/?event=register.home) (accessed on 22 December 2020). The search filters used were the following: “claims status: authorised”, “type of claim: Art. 14(1)(a) and Art. 13”, “EFSA Opinion reference: all”, “Legislation: all” [24].

### 2.2. Food Supplements Market Research

Second, a comprehensive search of the food supplements commercialized in the Spanish market, labelled with any of the approved health-related claims for folic acid, was carried out. This task was performed in two phases: the first one consisted of a preliminary search through the official online purchasing platforms used by 10 food establishments with a high market share in Spain and other e-commerce companies. Besides, an in situ search in the above-mentioned national establishments was performed. Information of both online and in situ searches was checked and verified by all authors and provided them a wide and detailed overview of the state of the art regarding food supplements marketed with health claims related to folic acid. Table S1 includes the designed template used to compile information about food supplements products and health-related claims included in their labeling, presentation, and/or advertisement.

### 2.3. Analytical Determination of Folic Acid

Third, the chemical analysis of the selected food supplements consisted in the determination of the content of folic acid. The term “folic acid” or “vitamin B_9_” includes vitamers with an equivalent biological activity. There are other terms in the literature that are interchangeably used to refer to folic acid, such as “folate(s)”, “folacin”, and “pteroylmonoglutamic acid” (PteGlu). The chemical structure of folic acid includes a pteridine ring, a residue of p-aminobenzoic acid, and a residue of glutamic acid, all of them linked by a methylene bridge and an amide bond. All the folate derivatives have this main chemical structure called “pteroylmonoglutamic acid” and differs from each other by their oxidation status, the substituents of the pteridine ring, and the number of glutamate residues that are linked to the p-aminobenzoglutamate residue by a peptide bond, the most frequent being mono-, penta- and hexa-glutamates. As folic acid is only synthesized by microorganisms and plants, humans depend on several dietary sources for its intake. Folic acid is naturally presented in food matrices as polyglutamates, which are not directly absorbable by the organism. These polyglutamates must be hydrolyzed in the intestinal mucosa by enzymes to obtain monomer units. Only one of these monomer units, i.e., tetrahydrofolate monoglutamate (THF-monoglutamate), is bioavailable and absorbable by the human organism. Once absorbed, THF-monoglutamate is transformed to 5-methyl-THF-monoglutamate (5-MTHF-monoglutamate), the biologically active vitamer.

The method for folic acid extraction was adapted from the one used by Morales et al. (2015) [26]. The determination of both vitamers was carried out by extraction in a buffer medium and quantification through High-Performance Liquid Chromatography (HPLC) with fluorescence detection for 5-MTHF-monoglutamate vitamer and UV–visible detection for pteroylmonoglutamic acid. In a Falcon tube, 1 g of each food supplement sample was weighed and 10 mL of NaH_2_PO4 · 2H_2_O phosphate buffer (100 Mm, pH = 4.4) was added. The resulting mixture was subjected to (1) agitation for 30 minutes in the dark and at a temperature of 80 °C in a magnetic stirrer (Selecta, Spain) and (2) subsequent sonication (30 minutes, t = 50 °C) in an ultrasonic bath (Selecta, Spain). These conditions were finally considered optimal to achieve the complete dissolution of the granules after testing other solvents. A methanol–distilled water (50:50) mixture, NaH_2_PO_4_/Na_2_HPO_4_ buffer (0.1 mol/L; pH = 9), and Na_2_HPO_4_ · 2H_2_O phosphate buffer (100 mM; pH = 7) were tested according to different extraction methods proposed by Matias et al. (2014) and Morales et al. (2015) [26,27]. These solvents, including the eventually selected one (NaH_2_PO4 · 2H_2_O phosphate buffer; 100 mM; pH = 4.4), were tested at different temperatures (0 °C, 50 °C, and 80 °C) and stirring times (30 and 60 min).

The resulting solution was centrifuged at 7000 revolutions per minute (rpm) for 15 minutes. The centrifuge used was a Universal 320 model (Hettich Zentrifugen, Kirchlengern, Germany). The supernatant liquid obtained was filtered through a Millex PVDF 0.45 µm filter into a vial, prior injection for identification and quantification by HPLC. Figure S1 shows the characteristics of the chromatographic equipment as well as the conditions used in the determination of each vitamer of folic acid.

#### Statistical Analysis

The analyzed food supplement samples contained one of the two following vitamers of folic acid: 5-MTHF-monoglutamate and pteroylmonoglutamic acid. Two batches of each sample were analyzed in triplicate. The data obtained were statistically evaluated through T Student’s t test, using α = 0.05 as the level of statistical significance. The software used for carrying out the above-mentioned statistical analysis was Statgraphics Plus 5.1.

## 3. Results and Discussion

### 3.1. Scientific Literature Review of Health-Related Claims Approved for Folic Acid in the European Regulation

According to the European Database “EU Register of nutrition and health claims made on foods”, there are nine claims currently approved in the European regulation: eight health claims and one “Reduction of disease risk” claim [24]. As it is shown in Table 1, the “Reduction of disease risk” claim is referred to supplemental folic acid intake and it is only allowed in the labeling, presentation, and/or advertising of food supplements. The other health claims are related to folate content in both foods and food supplements that are considered a “Source of folate” in accordance with the requirements established in the Annexes of Regulation (EC) Nº 1924/2006 and Regulation (EU) Nº 1169/2011 [18,19].

### 3.2. Food Supplements Market Research Results

A comprehensive search of the food supplements commercialized in the Spanish market was carried out. As it is shown in Table 2, 81 food supplements with health-related claims attributed to folic acid were found. The great majority (86.4%) was marketed through online supermarkets’ platforms and other e-commerce companies. All food supplements sold in physical supermarkets were adequately labeled in terms of health-related claims, whereas 14.3% of the food supplements marketed through online supermarkets’ platforms and e-commerce companies showed at least one health claim not included in the positive list established by the European Regulation (Regulations (EC) 1924/2006, (EC) No 432/2012, and (EU) No 1169/2011) [18,19,31]. Some examples of the unapproved health claims shown in the labeling, presentation, and/or advertisement of those food supplements were the following: “folic acid contributes to normal cognitive function”, “folic acid helps the protection of cells from oxidative stress”, “folates contribute to the maintenance of normal regulation of the organism”, and “folates contribute to the normal functioning of the nervous system”. This percentage (14.3%) is in line with the last value published by the SCIRI (12.8%) that reflects the alerts regarding food supplements in Spain mainly caused by an incorrect labeling, as it was previously mentioned.

### 3.3. Sample Selection for Chemical Analysis

Among the 71 food supplements correctly labeled (in terms of health-related claims), samples were selected for chemical analysis in the lab. The selection criteria were (1) food supplements directed to pregnant women, with a clear and unequivocal indication about this specific target population in their labeling, presentation, and/or advertisement, (2) the inclusion of the “Reduction of disease risk” claim approved for folic acid, and (3) the inclusion of other health claims related to vitamins and/or minerals that contribute to the maintenance of an adequate health status of pregnant women. All the above-mentioned claims had to be approved by the European regulation and included in the positive list.

The application of the first and second inclusion criteria resulted in a first selection of 12 food supplements, as they were directed to pregnant women and included the “Reduction of disease risk” claim referred to folic acid in their labeling, presentation, and/or advertisement. Taking into account the third inclusion criterion, and as it is shown in Table 3, four food supplements were selected that strictly met all the established inclusion criteria. These four food supplements were commercialized through online platforms and were dosed in sachets to be suspended in water. Interestingly, only two of them included health claims related to folic acid in addition to the “Reduction of disease risk” claim referred to this compound.

According to the information shown in the label, FS1 was the product with the highest content of folic acid (500 µg pteroylmonoglutamic acid/sachet), whereas FS4 declared the lowest one (200 µg 5-MTHF-monoglutamate/sachet). FS2 and FS3 samples had both a declared value of 400 µg pteroylmonoglutamic acid/sachet.

### 3.4. Analytical Determination of Folic Acid and Assessment of the Level of Compliance of Its Health-Related Claims

Immediately after reception, selected food supplement samples commercialized in a granular form (FS1, FS2, FS3, and FS4) were stored in a cool, dry place away from heat and direct light, as recommended in the package leaflet so to preserve the nutritional composition. Two batches of each sample (FS1, FS2, FS3, and FS4) were analyzed in triplicate in order to evaluate the appropriateness of the health-related claims of folic acid, that is, the compliance with the specific use conditions of each claim in the labeling, appearance, and/or presentation of food supplements. For the analysis, folic acid standards were kept and prepared following the recommended storage and handling conditions provided by the manufacturer, and reagent blanks were prepared regularly and measured together with the samples in order to monitor the trueness of the data obtained.

Figure S2 includes the chromatograms obtained in the analysis of folic acid vitamers (5-MTHF-monoglutamate and pteroylmonoglutamic acid).

To comply with the Regulation and to avoid consumers’ misleading, the content of folic acid in the analyzed samples should not deviate substantially from the declared values shown in the labeling. The analytical mean values of all samples were slightly higher than the declared value in the labeling. It is difficult that these products maintain the exact declared value during their shelf-life period due to the high degradation rate of folic acid, that depends on storage conditions (temperature, humidity, light exposure) and storage time. In all analyzed samples, the folic acid content of the batches 1 and 2 were similar, with no statistically significant differences.

Tolerance for nutrient values (vitamins and minerals) declared in the labeling of food supplements was consensually agreed upon by the European Commission and the representatives of the Member States, and a Guidance document was published for competent authorities for the control of compliance with EU legislation on Regulation (EU) No 1169/2011, Council Directive 90/496/EEC and Directive 2002/46/EC. It is important to note that this Guidance cannot be considered as an official interpretation of the European legislation, as this right is reserved to the relevant judicial authorities [32].

The application of tolerance covers one important factor related to food safety, as it sets the maximum contents of certain vitamins and minerals whose excessive intake could provoke important adverse effects [32]. According to the above-mentioned European Guidance, the values of folic acid declared for our samples met the requirements, as they did not deviate from the Range of Tolerance (RT) calculated for each food supplement (Table 4).

The eight health claims approved for folic acid (see Table 1) can only be declared in those products which are at least a “Source of folate” (Regulations (EC) No 1924/2006 and (EU) No 1169/2011), that is, a minimum content of 15% of the Nutrient Reference Value (NRV) of folic acid is supplied by 100 g of product (NRV of folic acid = 200 µg/100 g; 15% NRV = 30 µg/100 g) [18,19,31]. 

As it was mentioned in Section 3.3 and in Table 3, only the FS1 and FS2 samples included health claims related to folic acid. FS1 showed three health claims (“Folate contributes to maternal tissue growth during pregnancy“, “Folate has a role in the process of cell division”, and “Folate contributes to the normal function of the immune system”). FS2 included two health claims in its labeling, presentation, and/or advertisement (“Folate has a role in the process of cell division” and “Folate contributes to the normal homocysteine metabolism”). The “Reduction of disease risk” claim (“Supplemental folic acid intake increases maternal folate status. Low maternal folate status is a risk factor in the development of neural tube defects in the developing fetus”) can only be used in the labeling of those food supplements that provide a daily intake of 400 µg of folic acid. All samples (FS1, FS2, FS3, and FS4) reported the “Reduction of disease risk” claim in their labeling (see Table 3). According to the results of the present work, the analyzed food supplements met the legal requirements and could legitimately include the nine health-related claims of folic acid (eight health claims and 1 “Reduction of disease risk” claim) approved by the European regulation. Although the FS4 sample contained 200 µg folic acid/sachet, it fulfilled the requirement of the “Reduction of disease risk” claim, as its labeling advised pregnant women to take two sachets per day (that is, a daily intake of 400 µg folic acid).

Finally, in Table 5, we summarize the assessment process of the level of compliance of the health-related claims of folic acid in each food supplement sample that we carried out in the present work.

Further studies with a higher number of samples commercialized in other markets within the European Union are needed to allow a better picture of the state of the art of this issue.

## 4. Conclusions

Consumers deserve high-quality and safe food supplements that bear reliable information and fulfill the health effects promised by the health-related claims reported in their labeling. This is particularly important for pregnant women, who usually take food supplements with folic acid, for its crucial role in embryonic development and formation of the fetal tissues.

Our analytical results of folic acid were consistent with the declared values shown in the labeling of the corresponding food supplements and within the ranges of tolerance according to the European Guidance. Taking into account our analytical results, it can be concluded that all food supplement samples met the legal requirements (Regulations (EC) 1924/2006, (EC) No 432/2012 and (EU) No 1169/2011) for reporting the nine claims approved for folic acid (eight health claims and one “Reduction of disease risk” claim). Interestingly, all samples could include more health claims referred to folic acid than the ones already declared in their labeling; however, the manufacturers could have selected claims that they believe best address the particular situation and physiological conditions of pregnant women.

## Figures and Tables

**Table 1:** Health-related claims approved for folic acid according to the European regulation.

Model Claim	European Food Safety Authority (EFSA) Opinion Reference	
Health claims		
“Folate contributes to maternal tissue growth during pregnancy”	[28]	
“Folate contributes to normal amino acid synthesis”	[29]	
“Folate contributes to normal blood formation”	[28]	
“Folate contributes to normal homocysteine metabolism”	[28]	
“Folate contributes to normal psychological function”	[29]	
“Folate contributes to the normal function of the immune system”	[28]	
“Folate contributes to the reduction of tiredness and fatigue”	[29]	
“Folate has a role in the process of cell division”	[28,29]	
Use conditions: Food and food supplements which are at least a “Source of folate” according to the requirements established in the Annex to Regulation (EC) Nº 1924/2006		
Reduction of disease risk claim		
“Supplemental folic acid intake increases maternal folate status. Low maternal folate status is a risk factor in the development of neural tube defects in the developing fetus”		[30]
Use conditions: Food supplements that provide at least 400 µg of folic acid/day. The target population is women of child-bearing age, and the beneficial effect is obtained with a supplemental folic acid daily intake of 400 µg for at least one month before and up to three months after conception		

**Table 2:** Summary of the folic acid food supplements directed to pregnant women found in the Spanish market.

Source	Total Number	Food Supplements with Approved Health Claims Related to Folic Acid	Food Supplements with Unapproved Health Claims Related to Folic Acid
In situ purchase	11	11	0
Online purchase	70	60	10

**Table 3:** Application of the inclusion criteria to select food supplements samples containing folic acid that were subject to chemical analysis (FS1, FS2, FS3, and FS4).

	Inclusion Criterion 1 Target Population: Pregnant Women	Inclusion Criterion 2 “Reduction of Disease Risk” Claim Referred to Folic Acid	Inclusion Criterion 3 Health Claims Related to Vitamins and Minerals
Total	71	12	4
FS1	✓	✓	23 health claims Vitamins: B_6_, B_9_, B_12_, C, D Minerals: Cu, Fe, Zn, Ca, Mg, Se, I
FS2	✓	✓	4 health claims Vitamins: B_9_, D
FS3	✓	✓	2 health claims Minerals: Mn, Zn
FS4	✓	✓	5 health claims Vitamins: D Minerals: Fe, Ca, I

Inclusion criterion 1: food supplements directed to pregnant women; inclusion criterion 2: food supplements with the “Reduction of disease risk” claim approved for folic acid; inclusion criterion 3: food supplements with health claims related to vitamins and/or minerals which contribute to the maintenance of an adequate health status of pregnant women. (✓) symbol means requirement fulfilled.

**Table 4:** Assessment of the tolerance established for folic acid in the food supplements samples according to the Guidance document for competent authorities for the control of compliance with EU legislation with regard to the setting of tolerances for nutrient values declared in the labeling of food supplements.

Food Supplement Sample	Value Declared in the Labeling (µg/sachet)	Range of Tolerance (RT) (µg/sachet)	Analytical Value (µg/sachet)
FS1	500	399.60–750.60	509.55 ± 9.92
FS2	400	390.03–600.60	408.59 ± 9.47
FS3	400	396.25–600.60	429.52 ± 3.25
FS4	200	159.60–300.60	220.69 ± 9.09

**Table 5:** Summary of the assessment process of health-related claims of folic acid in each sample. FS1, FS2, and FS3 samples recommended in their labeling to take 1 sachet/day (500 µg folic acid/day for FS1 and 400 µg folic acid/day for FS2 and FS3), whereas the FS4 sample recommended 2 sachets/day (that is, 400 µg folic acid/day); all food supplements met the legal requirement established in the European regulation.

			Health Claims	Reduction of Disease Risk Claim
Sample	Declared Value (labeling)	Analytical Value (µg/sachet)	Legal Requirement to Use the Claims	Legal Requirement to Use the Claim
FS1	500 µg/sachet 1 sachet = 14 g	509.55 ± 9.92	Food supplements must be a “Source of folate”, that is, 15% of the Nutrient Reference Value (NRV) of folic acid is supplied by 100 g NRV of folic acid = 200 µg/100 g 15% NRV = 30 µg/100 g	Food supplements must provide at least 400 µg of folic acid/day.
FS2	400 µg/sachet 1 sachet = 4.02 g	408.59 ± 9.47		
FS3	400 µg/sachet 1 sachet = 2.3 g	429.52 ± 3.25		
FS4	200 µg/sachet 1 sachet = 6 g	220.69 ± 9.09		

## Supplementary Materials

The following are available online at https://www.mdpi.com/2072-6643/13/3/937/s1, Figure S1: Chromatographic equipment and HPLC conditions used in this study for the determination of folic acid vitamers, Figure S2: Chromatograms obtained for 5-MTHF-monoglutamate (measured by HPLC-fluorescence) and pteroylmonoglutamic acid (measured by HPLC-UV), Table S1: Designed template used to compile information from Spanish market research.

## References

[1] European Parliament and Council of the European Union (2002). Directive 2002/46/EC of the European Parliament and of the Council of 10 June 2002 on the approximation of the laws of the Member States relating to food supplements. Off. J. Eur. Union.

[2] European Food Safety Authority (EFSA) Food Supplements—Introduction. https://www.efsa.europa.eu/en/topics/topic/food-supplements.

[3] Domínguez Díaz L., Fernández-Ruiz V., Cámara M. (2019). The frontier between nutrition and pharma: The international regulatory framework of functional foods, food supplements and nutraceuticals. Crit. Rev. Food Sci. Nutr..

[4] Bureau Européen des Unions de Consommateurs (BEUC) (2016). Food Supplements. Challenges & Risks for Consumers.

[5] Funnell G., Naicker K., Chang J., Hill N., Kayyali R. (2018). A cross-sectional survey investigating women’ information sources, behaviour, expectations, knowledge and level of satisfaction on advice received about diet and supplements before and during pregnancy. BMC Pregnancy Childbirth.

[6] Jun S., Gahche J.J., Potischman N., Dwyer J.T., Guenther P.M., Suader K.A., Bailey R.L. (2020). Dietary Supplement Use and Its Micronutrient Contribution During Pregnancy and Lactation in the United States. Obstet. Gynecol..

[7] World Health Organization (WHO) (2016). Good Maternal Nutrition. The Best Start in Life.

[8] Koletzko B., Cremer M., Flothkötter M., Graf C., Hauner H., Hellmers C., Kersting M., Krawinkel M., Przyrembel H., Röbl-Mathieu M. (2018). Diet and Lifestyle Before and During Pregnancy—Practical Recommendations of the Germany-wide Healthy Start—Young Family Network. Geburtsh Frauenheilk.

[9] Meija L., Rezeberga D., Proper Maternal Nutrition during Pregnancy Planning and Pregnancy: A Healthy Start in Life Recommendations for Health Care Professionals—The Experience from Latvia Recommendations for Health Care Specialists; 2017. https://www.euro.who.int/en/health-topics/disease-prevention/nutrition/publications/2017/proper-maternal-nutrition-during-pregnancy-planning-and-pregnancy-a-healthy-start-in-life-2017.

[10] Ducker G.S., Rabinowitz J.D. (2017). One-Carbon Metabolism in Health and Disease. Cell Metab..

[11] Oliver E.M., Grimshaw K.E.C., Schoemaker A.A., Keil T., McBride D., Sprikkelman A.B., Ragnarsdottir H.S., Trendelenburg V., Emmanouil E., Reche M. (2014). Dietary Habits and Supplement Use in Relation to National Pregnancy Recommendations: Data from the EuroPrevall Birth Cohort. Matern. Child Health J..

[12] European Commission Food Supplements. https://ec.europa.eu/food/safety/labelling_nutrition/supplements_en.

[13] Brown B., Wright C. (2020). Safety and efficacy of supplements in pregnancy. Nutr. Rev..

[14] European Commission RASFF—Food and Feed Safety Alerts. https://ec.europa.eu/food/safety/rasff_en.

[15] European Commission RASFF Portal. https://webgate.ec.europa.eu/rasff-window/portal/?event=searchResultList&StartRow=1.

[16] National Food Institute—Technical University of Denmark (DTU) Many Danes Take Dietary Supplements although Few Need Them. https://www.food.dtu.dk/english/news/2016/03/many-danes-take-dietary-supplements-although-few-need-them?id=f9017469-c293-4f28-bd71-163a491a4ae8.

[17] German Federal Institute for Risk Assessment (BfR) German Test Suggests “Many” Vitamin Supplements Exceed Safe Dose Limits. https://www.nutraingredients.com/Article/2017/09/07/German-test-suggests-many-vitamin-supplements-exceed-safe-dose-limits?utm_source=copyright&utm_medium=OnSite&utm_campaign=copyright.

[18] European Parliament and Council of the European Union (2006). Regulation (EC) No 1924/2006 of the European Parliament and of the Council of 20 December 2006 on nutrition and health claims made on food. Off. J. Eur. Union.

[19] European Parliament and Council of the European Union (2011). Regulation (EU) No 1169/2011 of the European Parliament and of the Council of 25 October 2011 on the provision of food information to consumers, amending Regulations (EC) No 1924/2006 and (EC) 1925/2006 of the European Parliament and of the Council, and repealing Commission Directive 87/250/EEC, Council Directive 90/496/EEC, Commission Directive 1999/10/EC, Directive 2000/13/EC of the European Parliament and of the Council, Commission Directives 2002/67/EC and 2008/5/EC and Commission Regulation (EC) No 608/2004. Off. J. Eur. Union.

[20] Domínguez Díaz L., Fernández-Ruiz V., Cámara M. (2020). An international regulatory review of food health-related claims in functional food products labeling. J. Funct. Foods.

[21] Domínguez Díaz L., Dorta E., Maher S., Morales P., Fernández-Ruiz V., Cámara M., Sánchez-Mata M.C. (2020). Potential Nutrition and Health Claims in Deastringed Persimmon Fruits (*Diospyros kaki* L.), Variety ‘Rojo Brillante’, PDO ‘Ribera del Xúquer’. Nutrients.

[22] Getov I.N., Lebanova H., Grigorov E. (2011). Food supplements—marketing and health claims analysis. Arch. Balk. Med Union.

[23] U.S. Pharmacopeia (USP) (2016). Ensuring the Quality of Dietary Supplements. https://www.usp.org/sites/default/files/usp/document/about/public-policy/public-policy-dietary-supplements.pdf.

[24] European Commission EU Register of Nutrition and Health Claims Made on Food. http://ec.europa.eu/food/safety/labelling_nutrition/claims/register/public/?event=register.home.

[25] Agencia Española de Seguridad Alimentaria y Nutrición (AESAN) Informes del Sistema Coordinado de Intercambio de Información (SCIRI). http://www.aecosan.msssi.gob.es/AECOSAN/web/seguridad_alimentaria/subseccion/SCIRI.htm.

[26] Morales P., Fernández-Ruiz V., Sánchez-Mata M.C., Cámara M., Tardío J. (2015). Optimization and Application of FL-HPLC for Folates Analysis in 20 Species of Mediterranean Wild Vegetables. Food Anal. Methods.

[27] Matias R., Ribeiro P.R.S., Sarraguça M.C., Lopes J.A. (2014). A UV spectrophotometric method for the determination of folic acid in pharmaceutical tablets and dissolution tests. Anal. Methods.

[28] European Food Safety Authority (EFSA) (2009). Scientific Opinion on the substantiation of health claims related to folate and blood formation, homocysteine metabolism, energy-yielding metabolism, function of the immune system, function of blood vessels, cell division, and maternal tissue growth during pregnancy pursuant to Article 13(1) of Regulation (EC) No 1924/2006. EFSA J..

[29] European Food Safety Authority (EFSA) (2010). Scientific Opinion on the substantiation of health claims related to folate and contribution to normal psychological functions, maintenance of normal vision, reduction of tiredness and fatigue, cell division and contribution to normal amino acid synthesis pursuant to Article 13(1) of Regulation (EC) No 1924/2006. EFSA J..

[30] European Food Safety Authority (EFSA) (2013). Scientific Opinion on the substantiation of a health claim related to increasing maternal folate status by supplemental folate intake and reduced risk of neural tube defects pursuant to Article 14 of Regulation (EC) No 1924/2006. EFSA J..

[31] European Commission (2012). Commission Regulation (EU) No 432/2012 of 16 May 2012 establishing a list of permitted health claims made on food, other than those referring to the reduction of disease risk and to children’s development and health. Off. J. Eur. Union.

[32] European Commission Guidance Document for Competent Authorities for the Control of Compliance with EU Legislation on Regulation (EU) No 1169/2011, Council Directive 90/496/EEC and Directive 2002/46/EC with Regard to the Setting of Tolerances for Nutrient Values Declared on a Label. https://ec.europa.eu/food/safety/labelling_nutrition/labelling_legislation/nutrition-labelling_en.

