# Supplementary material for: Assessment of Health Claims Related to Folic Acid in Food Supplements for Pregnant Women According to the European Regulation
Source: Nutrients. 2021 Mar 14;13(3):937. doi: 10.3390/nu13030937 (PMC7998675; doi:10.3390/nu13030937)
Supplement: Supplementary file 1 [file nutrients-13-00937-s001.pdf]

5-MTHF-monoglutamate	*Pteroylmonoglutamic acid*
Equipment	***Equipment***
Pump: HPLC pump (model PU-1580; Jasco, Japan)<br>Degassing: degassing system (model BG-14, GASTORR; Flom Corporation, Japan)<br>Injector: auto-sampler (model AS-950; Jasco, Japan)<br>Detector: fluorescence detector (model FP-2020 Plus, Jasco, Japan)<br>Column: LiChrospher® 100 RP-18 endcapped (5 µm; LiChroCART® 250-4 HPLC-Cartridge; Merck, Darmstadt, Germany)<br>Software: CromaNec XP (Micronec, Spain)	Pump: HPLC pump (model Concept Series PU II; MSI; United Kingdom)<br>Injector: auto-sampler (model AS-1555-10; Jasco, Japan)<br>Detector: UV-visible detector (Spectra Series UV100; Thermo Separation Products, United States)<br>Column: LiChrospher® 100 RP-18 endcapped (5 µm; LiChroCART® 250-4 HPLC-Cartridge; Merck, Darmstadt, Germany)<br>Software: CromaNec XP (Micronec, Spain)
Conditions	***Conditions***
Room temperature<br>Mobile phase (A:B, 94:6): Solvent A: $NaH_2PO_4 \cdot 2H_2O$ (100 mM, pH = 4.4); Solvent B: acetonitrile for fluorescence detection<br>Flux: 1 mL/min (isocratic conditions)	Room temperature<br>Mobile phase (A:B, 94:6); Solvent A: $NaH_2PO_4 \cdot 2H_2O$ (100 mM, pH = 4.4); Solvent B: acetonitrile for UV-visible detection<br>Flux: 1 mL/min (isocratic conditions)

Figure S1. Chromatographic equipment and HPLC conditions used in this study for the determination of folic acid vitamers.

5-MTHF-monoglutamate

(a) Commercial standard

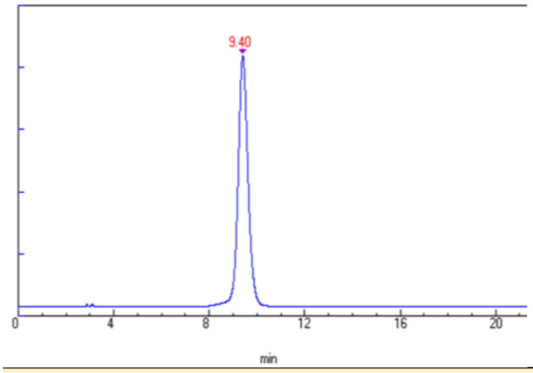

(b) Extract of FS4 sample

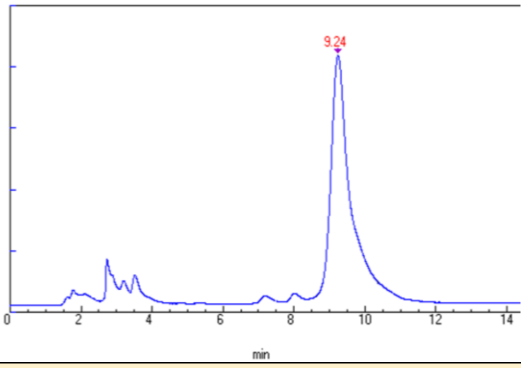

Pteroylmonoglutamic acid

(c) Commercial standard

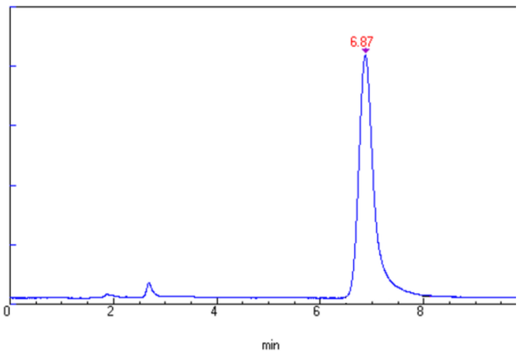

(d) Extract of FS1 sample

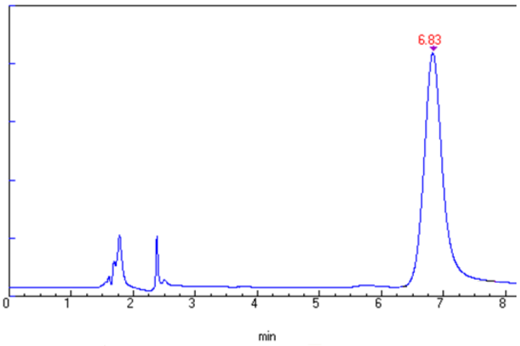

(e) Extract of FS2 sample

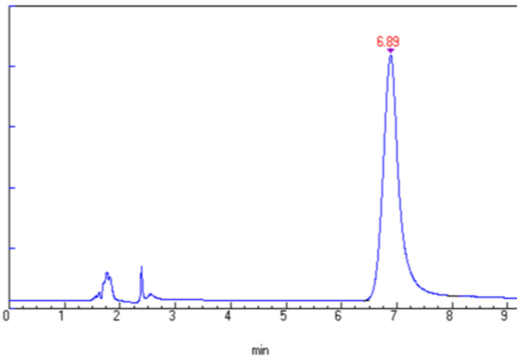

(f) Extract of FS3 sample

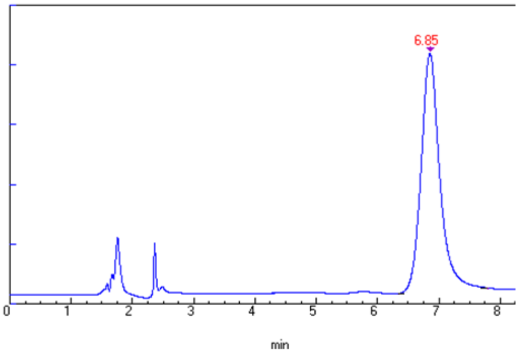

Figure S2. Chromatograms obtained for 5-MTHF-monoglutamate (measured by HPLC-fluorescence) and pteroylmonoglutamic acid (measured by HPLC-UV): (a) Commercial standard of 5-MTHF-monoglutamate; (b) Extract of FS4 sample containing 5-MTHF-monoglutamate; (c) Commercial standard of pteroylmonoglutamic acid; (d) Extract of FS1 sample containing pteroylmonoglutamic acid; (e) Extract of FS2 sample containing pteroylmonoglutamic acid; (f) Extract of FS3 sample containing pteroylmonoglutamic acid. Chromatographic conditions: see Figure S1.

Table S1. Designed template used to compile information from Spanish market research.

Food supplement product	Source	Does it contain folic acid?	Is it directed to pregnant women?	Does it include folic acid health-related claims?	Does it include other health claims related to vitamins and minerals?	Comments/remarks
	In situ/Online	YES/NO	YES/NO	YES/NO	YES/NO	
